# Supplementary material for: Clinical evaluation of the SD Biosensor SARS-CoV-2 saliva antigen rapid test with symptomatic and asymptomatic, non-hospitalized patients
Source: PLoS One. 2021 Dec 22;16(12):e0260894. doi: 10.1371/journal.pone.0260894 (PMC8694432; doi:10.1371/journal.pone.0260894)
Supplement: Attachment — Submitted filename: Response to Reviewers.docx [file pone.0260894.s002.docx]

**POINT-TO-POINT RESPONSE TO THE REVIEWER**

We thank the reviewers for their valuable comments, which we will address point-to-point below. The manuscript underwent significant revision thanks to the comments. The line numbers below correspond to the manuscript without track changes.

**Reviewers comments:**

**Reviewer #1**:

The manuscript is clearly written and presents public health relevant findings. It will benefit from minor revision in relation to the data presentation and interpretation in the public health context.

*Title: it would be helpful to specify in the title that this saliva test is for SARS-CoV2 antigen.*
**Specified**

*Abstract: clear and appropriate

Introduction: It would be valuable to introduce an applied epidemiology perspective here; such as
a) The manuscript discusses PPV, yet in public health, the NPV can be equally valuable. Especially in the context of self-tests being used by citizens prior to engaging in increased social interaction. Please elaborate on this perspective (useful reference: European Centre for Disease Prevention and Control. Considerations on the use of self-tests for COVID-19 in the EU/EEA – 17 March 2021. ECDC: Stockholm; 2021)*

***Comment: addressed in detail in results (Table 2) and discussion. Also as a study was running for 4 weeks and during that period the amount of positive people ie % PCR positivity changed the mean value was used to calculate NPV and PPV. Furthermore in the discussion scenarios with lowest and highest % PCR positivity figures were explored. (Lines 221-231)***

*b) the dependency of population prevalence. In the manuscript, only the prevalence among the tested population is used, though it would be insightful to understand what was the population COVID19 prevalence at the time of this study;*

***Clarified in results section (Lines 128-129)***

*c) Please describe what is known about access to testing in the described population of Rotterdam: how large is the estimated group that is unable to come to these XL-locations? This is relevant context in the discussion on the value of self tests.*
***Comment: it is difficult to know precisely, we can only estimate from the number of people having low socio economic status (SES) in Rotterdam. Several studies have shown that marginalised people often represent hard to reach population. Furthermore, self-testing is not only to reach marginalized people but also to encourage more frequent testing in any population. Some of these discussion points are included in the discussion.***

*Methods: Clearly written. It would be helpful to clarify why only 789 of the 816 included persons had a PCR result.*
**Clarified in Lines 84-85**

*Results:
- Define 'recent onset' (P4-L89) (from table 1, we deduce this must be <8 days, but this could be made explicit)*
**Comment: in this particular case (now line 125) recent onset is clarified in the bracket following the statement ie 2 days. However point taken and statement clarified throughout the manuscript.**

*- table 1:
Every time when "CT<=30" is printed in the table, this should be "CT<30"*
**Corrected: Categories changed slightly: Ct ≤ 25, Ct ≤ 30 and Ct >30**

*The numbers of CT>=30 and CT<30 among the NP AgRDT group should add up, yet they don't: 9+39=48, yet 49 (of 52) should have a positive PCR. Please check the data*

**Correction and comment: the categories were changes as detailed in the above point. The reason for the difference in total RDT positives and the total which has ct value is due to specificity of the RDT ie false positives detected by RDT. For clarification an extra row was added in the table.**

*The numbers of CT>=30 and CT<30 among the saliva AgRDT group should add up, yet they don't: 2+38=40, yet 41 (of 44) should have a positive PCR. Please check the data*
**Correction and comment: see comment above.**

*- Table 2: (page 7)
It is not clear why the N is larger than 556 in the columns of "0-3 days post onset" and "0-7 days post onset". It is my understanding that only 556 participants indicated an onset of symptoms.*
**Corrected and comment: indeed confusingly the total population size was displayed not the concerning true positives/true negatives which are necessary for calculating the sensitivity/specificity.**

*The row
- Table 2 (page 8)
Here too: It is not clear why the N is larger than 556 in the columns of "0-3 days post onset" and "0-7 days post onset". It is my understanding that only 556 participants indicated an onset of symptoms.
Same on page 9*
**Clarified: However 556 people stated presence of symptoms only 545 provided date of onset. This was clarified in the text (Line 126)**

*Discussion:
Clear focus chosen on the discussion topics. What is missing, is a perspective on public health consequences of the PPV and the NPV found in this study: it would not be inappropriate to indulge in a 'what if' scenario, exploring how the saliva test would perform if used widely in the given population of Rotterdam, given the estimated COVID19 prevalence at that time.*

**Added: Lines 221-231** *Lastly, it would be of interest to elaborate a bit on the risk of aerosolisation while taking the saliva samples, and discuss how that would compare to the other tests used in this study.*

**Edited*:* the above mentioned points were addressed as much as possible in the discussion (Lines 212-215).**


**Reviewer #2***:*

Review of “Clinical evaluation of the SD Biosensor saliva antigen rapid test with symptomatic and asymptomatic, non-hospitalized patients”. Short article with promising results. Methods section is very clear. The results could be better displayed as far as I am concerned. Also, the statistical comparison that is made to compare sensitivity estimates by time since symptom onset needs to be looked at more closely. Furthermore, I found it very encouraging that the saliva antigen test was able to detect 97% of the positive viral cultures. This is in my opinion a very convincing result to implement this easy-to-use test as a public health tool for in particular health care workers and for the general public for SARS-CoV-2 and potentially beyond for other infectious diseases as well for example influenza.

Major points: *I find the results section too brief. I would like to see clearer sentences where it is made explicit which two tests are being compared and show how the sensitivity was estimated. For example, "of the XX samples that tested positive with PCR saliva, XX tested positive with the saliva antigen test (XX/ XX = XX sensitivity)." I find it difficult at this point to determine from the text which tests were compared and how sensitivity was estimated.*
**Result section was rewritten and restructured and part of Table 2 removed as I believe that was confusing and redundant information.**

*To me the main finding of this study is that the saliva RDT detected all but one positive virus cultures. Assuming virus culture is the best indicator of infectiousness, the saliva RDT is in this study the best test to detect infectious persons, and would therefore be very suitable for at home use, and therefore be an excellent public health tool to stop transmission. I think this finding deserves a better location in the results and discussion section.*

**Comment: this was highlighted better both in results and discussion. In this study we did not culture from nasopharyngeal swabs however we did that in our previous studies and similar results were achieved meaning by Ct cut-off and days post onset ie early phase more infectious. In this current study days post onset did not result in increase due to the above detailed skewed dataset.**

*Line 100: Overall performance of saliva was inferior to these other two tests. But comparing one test to another will always result in an inferior test, right? There is always going to be one sample, which was tested positive in the bench mark test, that will be negative in the evaluated test? Also, inferior in finding what? RNA or samples with infectious potential? The saliva test found more of the samples that tested positive in viral culture than the nasopharyngeal test, right? In that sense, the saliva test is superior over the nasopharyngeal test finding samples that positive with positive culture.*
**Comment: Indeed most comparisons going to be inferior and in this case well expected as we compared a very sensitive molecular test to a serological test which never can be as sensitive as the molecular. It was inferior in performance to detect every RT-PCR or nasopharyngeal RDT positive person but the reviewer is right pointing out the additional value of detecting infectious individuals. Real value does come from detecting these people. Section was rephrased to reflect this (Lines 165-173 and 195-200).**

*Table 2: I am confused about the N-values. In total, there were 789 samples tested. Of these, 62 were PCR-positive, 52 with the NP Ag RDT, and 44 with the saliva Ag RDT. The sensitivity estimates can therefore never be estimated with the numbers I am seeing in this table. I would provide the actual numbers from which the sensitivity estimates were estimates in the parentheses instead of one N number, for example: 79.2% (XX/XX)*

**Correction and comment: Numbers were indeed confusing therefore table was edited to make it clearer.**  *“Analysis by days post onset did not result in higher sensitivities”. How was this concluded? Looking at table 2, I see percentages for the group 0-3 days post onset and the group 0-7 post onset. For a proper comparison, one would have to make two different groups, for example comparing samples taken 0-3 days after symptom onset, and samples taken 4-7 days post symptom onset.*
**Correction and comment: in previous studies big difference was usually seen between people who had symptom onset 0-3 days vs 0-7. In this population/study we did not see this due to the uneven split of the population who participated in the study and the comment refers to this fact. It was made clear both by the presented numbers in Table 2 (48 vs 38 people with symptom onset 0-7 days vs 0-3 days) and in the text (in abstract and Line 151-153)**

*Minor points:
Line 47: People were either tested because of respiratory symptoms or because of being a contact of an individual with a confirmed SARS-CoV-2 infection as mentioned in line 47/48. Could you provide information on the number of samples that were from contact persons, perhaps in Table 1?*

**Answer: this specifically was not recorded in the questionnaire, one can assume that many of the asymptomatic people were contacts**.

*Line 62: This sentence requires rewriting.*

**Comment: sentence was rephrased (Line 83-85).**

*Line 98: To me, this summary statistic is redundant and without meaning.*

**Comment: whole result section was rewritten and restructured.**

*Line 99: Sensitivity increased to 88.6, but where is it increasing from?*

**Comment: sentence was rephrased. Increase from 63%, the overall sensitivity.**
